# Inventory of Sources of Stress During Medical Education - Further Validation

**DOI:** 10.1192/j.eurpsy.2022.1784

**Published:** 2022-09-01

**Authors:** M. Carneiro, A. Macedo, E. Loureiro, M. Dias, F. Carvalho, D. Telles Correia, F. Novais, C. Barreto Carvalho, C. Cabacos, D. Pereira, P. Vitória, A. Araújo, A.T. Pereira

**Affiliations:** 1 Centro Hospitalar e Universitário de Coimbra, Department Of Psychiatry, Coimbra, Portugal; 2 Faculty of Medicine of University of Coimbra, Institute Of Psychological Medicine, Coimbra, Portugal; 3 Coimbra Institute for Biomedical Imaging and Translational Research, -, Coimbra, Portugal; 4 Porto University, Faculty Of Medicine, Porto, Portugal; 5 Coimbra University, Faculty Of Medicine, Coimbra, Portugal; 6 Lisbon Medical University, Psychiatry, Lisbon, Portugal; 7 Lisbon University, Faculty Of Medicine, Lisbon, Portugal; 8 University of Azores, Department Of Psychology, Ponta Delgada, Portugal; 9 University of Beira Interior, Department Of Psychology And Education, Covilhã, Portugal

**Keywords:** burnout, medical students, Inventory of Academic Sources of Stress in Medical Education, confirmatory factor analysis

## Abstract

**Introduction:**

The Inventory of Academic Sources of Stress in Medical Education (IASSME) evaluates the presence and intensity of the main sources of academic stress for Portuguese Medicine students in five dimensions: Course demands/CD, Human demands/HD, Lifestyle/LS, Academic competition/AC, and Academic adjustment/AA.

**Objectives:**

To further validate the ISSME using Confirmatory Factor Analysis and to analyze the psychometric properties of a new version including additional sources of stress.

**Methods:**

Participants were 666 Portuguese medicine (82.6%) and dentistry (17.4%) students (81.8% girls); they answered an online survey including the ISSME and other validated questionnaires: Maslach Burnout Inventory – Students Survey (MBI-SS) and Depression Anxiety and Stress Scales (DASS).

**Results:**

Confirmatory Factor Analysis showed that the second order model composed of five factors (the original structure by Loureiro et al. 2008), but excluding item 11 (loading=.371), presented good fit indexes (χ2/df=3.274; RMSEA=.0581, p<.001; CFI=.917; TLI=.904, GFI=.919). The Cronbach’s alfas were α=.897 for the total and from α=.669 (F2-HD) to α=.859 (F1-CD) for the dimensions. The expanded version, including two additional items related to lack of interest in medicine/dentistry (F6, α=.543) and two additional COVID-19 stress-related-items (F7, α=.744) also showed acceptable fit indexes (χ2/df=3.513; RMSEA=.061, p<.001; CFI=.88.; TLI=.866, GFI=.892). This new version’s α was of .896. Pearson correlations between ISSME and the other measures were significant (p<.01) and high: >.55 with DASS and >.50 with MBI-SS. Girls presented significantly higher ISSME scores. F6 score was significantly higher in dentistry students.

**Conclusions:**

This further validation study underlines that IASSME presents good validity (construct and convergent) and reliability.

**Disclosure:**

No significant relationships.

